# Cone beam computed tomography-guided online adaptive radiation therapy: Clinical implementation in breast and axillary target volumes

**DOI:** 10.1016/j.ctro.2025.101086

**Published:** 2025-11-27

**Authors:** Angelique R.W. van Vlaenderen, Judith G. Middelburg-van Rijn, Koen J. Nelissen, Karin N. Goudschaal, Lars ter Beek, Jessica van der Himst, Cassey E. Glebbeek, Lisette M. van Maurik, Amber L. Bakker, Nina Bijker, Joost J.C. Verhoeff, Ben J. Slotman, Anna Dinkla, Wilko F.A.R. Verbakel, Desirée H.J.G. van den Bongard, on behalf of the BREAST-ART group

**Affiliations:** a Amsterdam UMC Location Vrije Universiteit Amsterdam, Radiation Oncology, Amsterdam, the Netherlands; b Cancer Center Amsterdam, Cancer Treatment and Quality of Life, Amsterdam, the Netherlands; c Amsterdam UMC Location University of Amsterdam, Radiation Oncology, Amsterdam, the Netherlands; d Varian Medical Systems, Inc., Radiotherapy Solutions, Palo Alto, USA; e Cancer Center Amsterdam, Cancer Biology and Immunology, Amsterdam, the Netherlands

## Abstract

**Background and purpose:**

Daily differences in breast contour and arm positioning can result in frequent re-positioning and offline re-planning in postoperative breast radiotherapy (RT). Online adaptive radiotherapy (oART) enables daily adaptation of the radiotherapy treatment plan. This study aimed to implement oART in breast and axillary target volumes to eliminate the need for repositioning and replanning.

**Materials and methods:**

Patients referred for postoperative partial breast irradiation (PBI) and whole breast irradiation (WBI)/post-mastectomy radiotherapy (PMRT) with or without axillary levels I-II (5x5.2 Gy) or I-IV (15x2.67 Gy) were included. On-couch treatment times were evaluated as well as dosimetry, target volume, acute toxicity, and in-house satisfaction questionnaires.

**Results:**

Seventy-nine patients (n = 66 PBI/WBI/PMRT, n = 13 WBI + axillary levels I-II/I-IV) were treated with postoperative RT (465 fractions). On-couch median time ranged from 14.5 (range: 10.1–29.2) minutes for right-sided WBI to 25.8 (range: 22.6–36.8) minutes for left-sided WBI + axillary levels I-II. All adapted treatment plans met the D98% ≥ 95 % coverage criteria for the target PTV. Relative breast CTV volumes for the adapted plan decreased in fraction 1 and increased in fractions 2–5 compared to the CTV volume on the planning CT. Toxicity was mainly grades 0 and 1 and patient satisfaction was good.

**Conclusion:**

Implementation of oART was feasible in all breast target volumes with acceptable treatment duration, only mild acute toxicity, favorable patient-reported comfort, and dosimetry that met clinical standards. Future work will focus on the implementation of oART in RT boost indications, and selection of breast cancer patients who will benefit most from oART.

## Highlights

• Online adaptive radiotherapy was implemented for breast and axillary target volumes.
• The on-couch median time ranged from 14.5—25.8 minutes over all indications.
• All adapted treatment plans met the D98% ≥ 95 % coverage criteria for the target PTV.
• An RTT-only workflow was clinically implemented.
• Manual adaptations were most needed for indications with axillary target volumes.

## Introduction

Postoperative radiotherapy (RT) may be indicated in breast cancer patients treated with breast-conserving surgery or mastectomy to decrease the risk of locoregional recurrence and improve survival.[1,2] In standard image-guided radiotherapy (IGRT) workflow, reference skin marks and laser lines are used, followed by a registration of the cone-beam CT (CBCT) with the planning CT (pCT) and a subsequent couch shift to optimally position the patient. However, accurate positioning of the target can be challenging due to the non-rigidity of the breast, tumor bed and the axillary lymph nodes (LN) that move independently from the breast and remaining body deformations.[3] Interfraction variations in the breast and tumor bed can be caused by post-operative seroma, breast edema, or different breathing patterns.[4] In addition, arm positioning variations can influence the position of the breast and axillary LN as postoperative pain and shoulder mobility may vary throughout the course of RT, causing reproducibility of the arm position to be challenging.[5] In-house data from a 6-month period showed that multiple CBCTs were needed to correct the patient position in at least one fraction during the RT-course in 59 % of the patients, corresponding to 18 % of all fractions (see Supplementary Materials, Appendix A). In addition, if re-positioning is not sufficient and the breast and LN clinical target volume (CTV) on the CBCT cannot be matched within the planning target volume (PTV) of the RT pCT, a repeat pCT and a new plan are needed. Based on in-house data collected from 2018 to 2022, 9 % of local breast radiotherapy cases required an offline re-planning and even 15 % in the case of locoregional breast RT.[6] With the implementation of ultra-hypofractionated schedules (5x5.2 Gy) in 2020, it has become even more important to switch as quickly as possible to a new treatment plan.[7] However, the offline re-planning process results in excess treatment burden for the patient and demands additional commitments from personnel and the logistics processes at the departments.

Due to technological advances, a CBCT-guided oART system (Ethos, Varian Medical Systems, Inc., Palo Alto, CA) that adapts and reoptimizes the treatment plan to the daily anatomy based on a CBCT is now a viable option.[8] In case of oART, interfractional anatomical variations no longer have an influence on the treatment plan and additional time for repositioning of the patients, extra CBCT’s, or offline re-planning is no longer needed. This can improve dosimetry of PTV and organs at risk (OAR) as shown in recent studies of oART in accelerated partial breast irradiation.[9,10] Recently, we showed that oART was feasible in 20 patients treated with right-sided whole breast irradiation (WBI). Target coverage was improved and OAR dosimetry was equivalent compared to the scheduled plan.[6] Despite these advantages, oART workflows may take longer than standard IGRT workflows and the introduction of oART requires a radiation oncologist (RO) and/or medical physics expert (MPE) to be present during the oART treatment procedure. In this study, we evaluated the implementation of oART in additional breast targets including left-sided WBI, axillary LN, and partial breast irradiation (PBI).

## Methods

### Study design and patient inclusion

The BREAST-ART trial (NCT05727553) is a single-arm prospective trial, approved by the Amsterdam UMC ethics committee (IRB 2021.0624). Eligible patients were treated between June 2022 and March 2025 at the Amsterdam UMC department of Radiation Oncology or at its satellite location. Patients were referred for PBI or WBI/post-mastectomy radiotherapy (PMRT) with/without axillary LN levels I-II or I-IV. The primary objective of this study was to evaluate the feasibility and duration of the oART workflow. Secondary objectives were to evaluate dosimetry, patient satisfaction, and treatment-related toxicity.

### Target definition and pre-treatment planning

CTVs were defined for PBI, WBI, PMRT, and axillary LN I-II or I-IV, and contoured according to the ESTRO guidelines.[11] The PTV-margin was 5 mm, and all targets were cropped 5 mm below the body contour. In patients with a thin chest wall, PTV was cropped 3–4 mm below the body. OARs were the contralateral breast, heart, and both lungs.

WBI/PMRT used a 4-beam tangential IMRT-technique. For WBI/PMRT + LN I-II and LN I-IV indications, a fifth field was added. This was followed by optimization to a 10-field IMRT-technique over 187-217° for WBI/PMRT + LN I-IV. For PBI, a VMAT technique with 2 half arcs was used. For each indication, a planning template was created based on the target goals for PTV D98% ≥ 95 %, D2% ≤ 107 % and Dmean between 99–101 % and OAR constraints for the heart (Dmean), contralateral breast (Dmean), lungs (Dmean) and the ipsilateral lung (V5Gy and V20Gy). These goals and constraints are according to the Dutch consensus on evaluation parameters based on the ICRU 83 guidelines and Quantec data, respectively.[12,13] In addition, a virtual bolus was used for all indications to improve plan robustness. Additional details on beam setup, use of virtual boluses, and planning templates are described in the supplementary materials, Appendix B. Based on this setup, a reference treatment plan (TP_R_) was automatically generated in the Ethos system using Ethos treatment planning version 1.1 for the first 11 patients, version 1.1MR1 for the subsequent 60 patients, and version 2.0 for the final 8 patients.

### Online Adaptive Treatment procedure

**Fig. 1:**
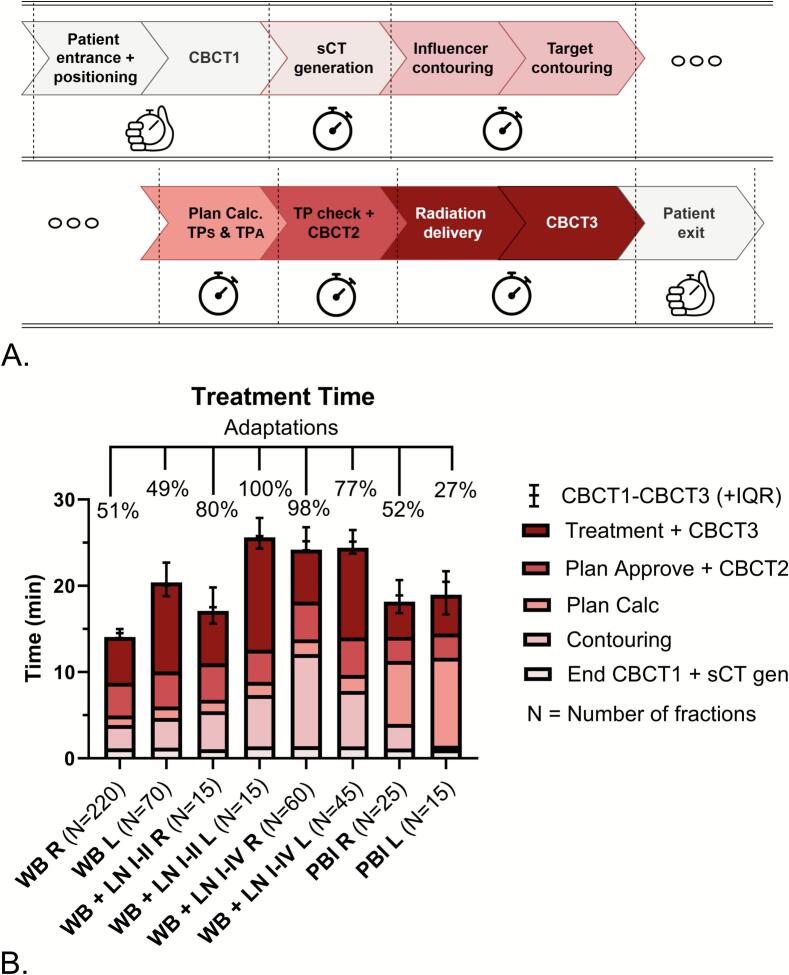
Treatment workflow and duration. A) Overview of the time segments of the workflow. B) Overview of the median on-couch treatment time for each time segment of the workflow for all indications including IQR for the CBCT1-CBCT3. On the X-axis each indication and in brackets the number of fractions. The percentage of fractions with manual adaptations of the CTV breast/chest wall/partial breast and lymph nodes if applicable are visualized per indication on top of the graph. Left-sided indications were treated using vDIBH. PBI is planned with VMAT. Time data for the segment part ‘treatment + CBCT3′ were missing in 9/220 WB R fractions, 2/55 WB L fractions, 1/60 WB + LN I-IV R fractions, and 2/45 WB + LN I-IV L fractions. Abbreviations: CBCT = cone beam computed tomography, sCT = synthetic CT, TP_S_ = scheduled treatment plan, TP_A_ = adapted treatment plan, TP = treatment plan, WB = whole breast, LN I-II/IV = axillary lymph nodes levels I-II/I-IV, PBI = partial breast irradiation, R = right-sided, L = left-sided vDIBH = voluntary deep inspiration breath hold.

The oART workflow is shown in Fig. 1A and has been described previously.[6] Dependent on the RT indication and location, no or one anteriorly applied skin mark for laser alignment was used to position the majority of patients followed by CBCT1. During the course of the study, the majority of our Ethos systems were upgraded with a Hypersight (Varian) CBCT imaging system enhancing the image quality.[14] All workflow developments are summarized in supplementary materials, Appendix C. After CBCT1, a deformable image registration from the pCT to CBCT1 generated a synthetic CT (sCT), used to propagate the influencer structure (equal to CTV Breast) and the target (CTV Breast) and OAR contours. The sCT has original pCT HUs and is used in the background for dose calculation, but it is not shown. The influencer structure should aid the propagation, but was used only in the first 17 WBI patients, since this resulted in worse contour propagation.[6] If necessary, the propagated structures were manually adapted by the radiotherapy technologists (RTTs), supervised by the RO. Following earlier results, no adaptations were made in the 2 most cranial/caudal slices of the CTV.[6] Hereafter, a scheduled treatment plan (TP_S_) and an adapted treatment plan (TP_A_) were calculated using the sCT, but shown on CBCT1. The TP_S_ is the TP_R_ recalculated onto the sCT (i.e. daily anatomy) while the TP_A_ is a reoptimized plan on the sCT using the planning template. Based on the dosimetric parameters and dose distribution, TP_A_ was typically selected. Subsequently, a second CBCT2 verified the patient’s positioning, with couch shifts applied as necessary, followed by treatment delivery and CBCT3. Quality assurance by performing a secondary dose check was conducted using Mobius software (Varian).

An RTT-only workflow was developed for WBI, PMRT, and PBI in which the RO and MPE were only present during the first fraction of treatment and on call for the remaining fractions according to protocol. Offline dose check was performed daily by the RO.

Right-sided breast cancer patients were treated in free-breathing, while left-sided cancer patients were treated using voluntary deep inspiration breath-hold (vDIBH) to minimize cardiac dose.[15] vDIBH was guided by lasers projecting on a small Styrofoam block (8x8 cm) on the patient’s upper abdomen. The block had three lines spaced 1.5 cm apart (3 cm total) of which one best fitting could be used for reference. The patient was instructed by the RTTs to take a DIBH of 20–25 s. RTTs manually started the radiotherapy delivery if the laser matched the correct position (i.e. best fitting reference line) on the block and stopped if the laser moved above or below the line.

### Data collection and statistics

The on-couch treatment time (i.e. from CBCT1 to CBCT3) was extracted from the DICOM data using a MATLAB (version R2021a) script. The time of entering and exiting the treatment room was manually logged by RTTs. Dosimetric data for each clinical goal was extracted from the DVH for each TP_R_, TP_S_, and TP_A_. The Wilcoxon signed ranks test was used for statistical comparison (*P* < 0.05) between TP_A_ and TP_S_ for the CTV and PTV D98%. Target volumes were extracted from each TP_R_ and delivered TP (TP_A_ or TP_S_). Volume differences between TP_R_ and fractions 1 and 2, and 2 and 5 to the related TP_R_ were compared using the paired samples *t*-test (*P* < 0.01). Performed couch shifts were extracted from the Ethos software and tested for correlation with time using Spearman’s rank correlation *(P* < 0.05).

Toxicity was evaluated using the Common Toxicity Criteria Adverse Events version 5.0 at baseline (post-surgery/pre-radiotherapy), and at 1 and 3 months after the last radiotherapy treatment fraction.[16] Patient’s satisfaction and experience was assessed via an in-house developed questionnaire completed after the final fraction, using a 4-point Likert scale (Supplementary materials, Appendix G).

## Results

### Patient and treatment characteristics

This study included 79 female patients: 53 were treated at Amsterdam UMC (VUmc) Department of Radiation Oncology and 26 at the Flevo Hospital satellite site. Patient and treatment characteristics are summarized in Table 1A, Table 1B.

**Table 1A:** Patient and tumor characteristics.

**Number of patients**	79
**Median age (range)**	63 (33–86) years
**Median time from pCT to RT**	15 (5–48) days
**Tumor Type**	
Invasive breast cancer	68
Ductal carcinoma in situ	10
Occult breast cancer	1
**Tumor Stage**	
Stage 0	10
Stage I	32
Stage II	33
Stage III	4
Stage IV	0

**Table 1B:** Radiotherapy characteristics.

**5x5.2 Gy (total dose 26.0 Gy)**	Number of patients		Number of fractions		Median (range) MU TP_R_	Median (range) CTV volume TP_R_ [cm^3^]	
	Right	Left	Right	Left		WB/CW/PBI	LN
WBI/PMRT*	44	14	220	70	1533 (1200–2049)	803 (82–2113)	−
PBI	5	3	25	15	1050 (813–1261)	64 (36–141)	
WBI + LN I-II	3	3	15	15	2002 (1664–2211)	610 (368–1580)	105 (84–128)
**15x2.67 Gy (total dose 40.05 Gy)**							
WBI/PMRT + LN I-IV**	4	3	60	45	1441 (1340–1661)	763 (424–1158)	188 (142–245)

### Treatment procedure and duration

On-couch treatment time (CBCT1-CBCT3) and frequency of manual adaptations per RT indication are illustrated in Fig. 1B. The median (range) duration ranged from 14.5 minutes (10.1–29.2) for right-sided WBI/PMRT to 25.8 (22.6–36.8) minutes for left-sided WBI/PMRT + axillary level I-II including vDIBH. In the latter, 100 % of fractions were manually adapted. The minimum amount of manual adaptations was 27 % for left-sided PBI. Due to incomplete time data on room entry/exit, the total treatment time is not illustrated but available in the supplementary materials, Appendix D, along with a numeric overview of the time segments. Absolute couch corrections based on CBCT1 and CBCT2 registrations just before treatment averaged 0.16 (SD: 0.14), 0.14 (SD: 0.13), and 0.10 (SD: 0.10) cm for vertical, longitudinal, and lateral directions, respectively. The mean length vector was 0.27 (SD: 0.16) cm and showed a very weak significant positive correlation with time (ρ(465) = 0.196, *P* < 0.001). Additional data can be found in the supplementary materials, Appendix E. In total, 135 RT fractions (29 %) were performed RTT-only, consisting of 95 fractions in right-sided WBI/PMRT, and 40 fractions in left-sided WBI/PMRT.

### Dosimetry

**Fig. 2:**
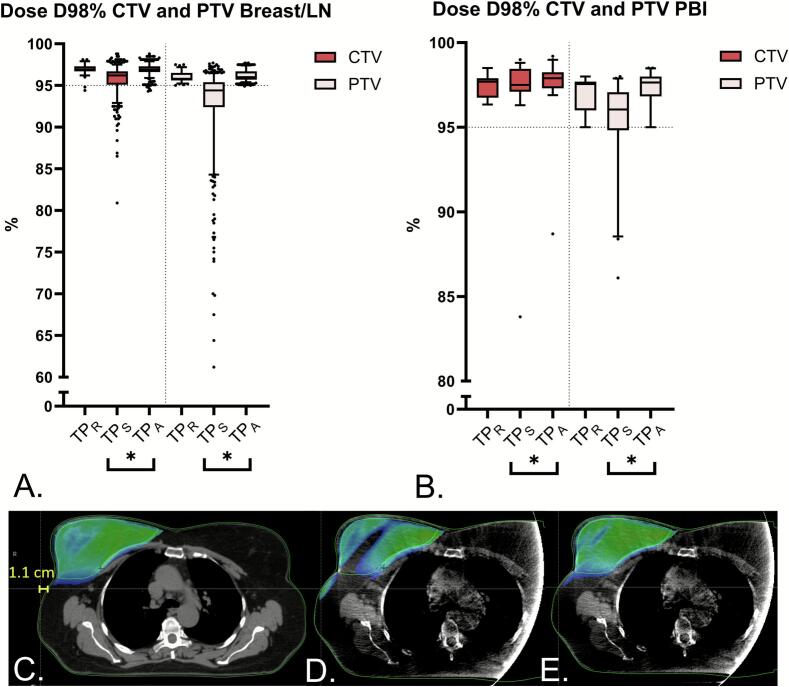
Target dosimetry. (A-B) D98% of the CTV and PTV of the TP_R_, TP_S_ and TP_A_. A) Breast targets of all patients excluding PBI (N = 71). B) Partial breast targets of PBI patients (N = 8). In both Figures A and B the middle line in the boxes indicates the median value while the borders of the boxes indicate the 25th and 75th percentile. The whiskers denote the 5th and 95th percentile. Star symbol indicates a significant difference between TP_S_ and TP_A_ (P < 0.05). (C-E) Example of C) a TP_R_ on the pCT, D) an underdosed TP_S_ on the first CBCT, and E) an adequately dosed TP_A_ on the first CBCT. Dose range is 95 %-109 %. Visualized in yellow the extent of the body contour difference between the CBCT and the pCT in the shoulder region. Abbreviations: CTV = clinical target volume, PTV = planning target volume, LN = axillary lymph nodes levels I-II/I-IV, PBI = partial breast irradiation, CBCT = cone beam computed tomography, TP_R_ = reference treatment plan, TP_S_ = scheduled treatment plan, TP_A_ = adapted treatment plan.

Significant dosimetric difference between TP_S_ and TP_A_ for both CTV (*P* < 0.001) and PTV (*P* < 0.001) targets were observed for local and locoregional patients (Fig. 2A), and for PBI patients (CTV *P* = 0.015, PTV *P* < 0.001) (Fig. 2B). All TP_R_ and TP_A_ reached ≥ 95 % coverage for PTV breast/LN and PBI, compared to only 37 % and 73 % in TP_S_, respectively. For CTV breast/LN, all TP_R_ and TP_A_ reached ≥ 95 % coverage, except for the TP_R_ (and 5/20 accompanying TP_A_) in two patients whose CTV was cropped less than 5 mm below the body contour (Table 1B). In comparison, only 80 % of the TP_S_ achieved this coverage. For CTV PBI, all TP_R_, TP_A_, and TP_S_ reached ≥ 95 % coverage, except for one fraction where the CTV PBI was mistakenly not cropped below the body. Fig. 2C–E shows an example of an underdosed CTV breast on TP_S_ and its adequately dosed TP_A_. Extended dosimetric data can be found in the supplementary materials, appendix F. No additional statistical tests were executed on dosimetry between TP_S_ and TP_A_ because only minimal differences were observed.

### Volumetric data

**Fig. 3:**
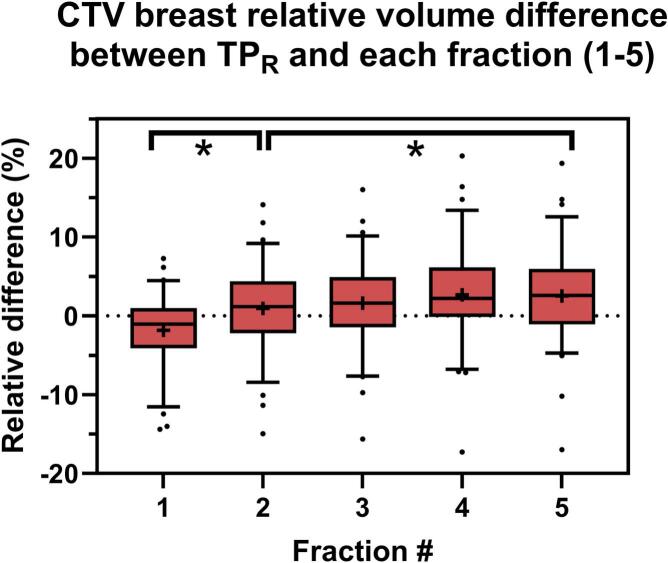
CTV breast volume change. Relative volume difference between each fraction and the accompanying TP_R_ of the CTV breast in 71 patients. The middle line in the boxes indicates the median value and the plus mark the mean value. The borders of the boxes indicate the 25th and 75th percentile, while the whiskers denote the 5th and 95th percentile. Star symbols indicate significant differences between indicated fractions (P < 0.001). Abbreviations: CTV = clinical target volume, TP_R_ = reference treatment plan.

The relative volume of the breast CTV decreased from the planning CT to the first fraction with a mean of 1.8 % (SD: 4.6) while, after the first fraction, it increased compared to the planning CT volume with 0.9 % (SD: 5.3) in fraction 2 and 2.5 % (SD: 5.7) in fraction 5 (N = 71 patients) (Fig. 3). The paired sample t-test between volume differences of fractions 1 and 2 compared to TP_R_ and 2 and 5 compared to TP_R_ showed a significant difference t(65) = 7.0, P= 0<.001 and t(65) = 3.7, P= 0<.001, respectively.

### Toxicity

**Fig. 4:**
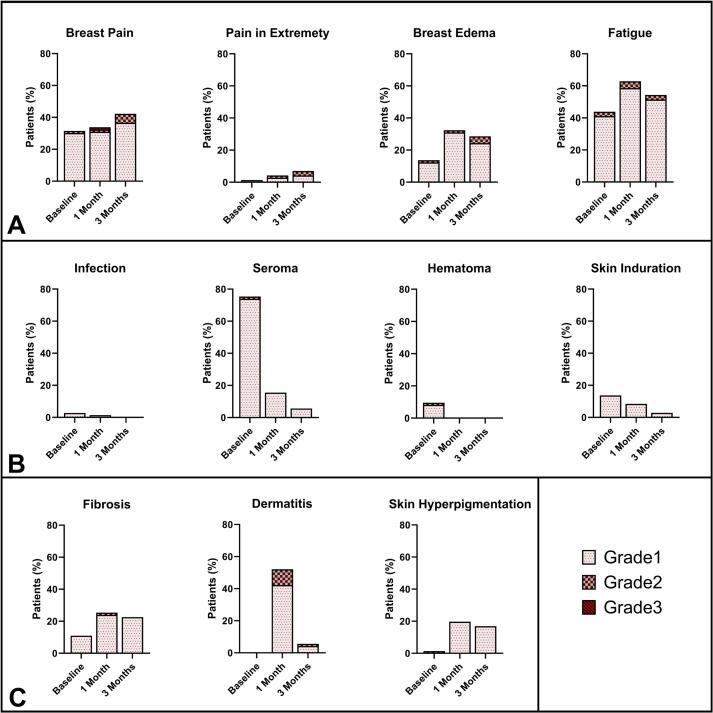
Acute toxicity. Acute toxicity at baseline (post-surgery/prior to radiotherapy, N = 73), and 1 (N = 71) and 3 months (N = 71) after the last radiotherapy treatment fraction of all patients. A) radiation therapy- and surgery-associated toxicities, B) surgery-associated toxicities, C) radiation therapy-associated toxicities.

Acute toxicity was predominantly graded 0–1 (Fig. 4). Most frequently reported surgery- and radiotherapy-associated toxicities were breast pain, breast edema, and fatigue. Most frequently reported radiotherapy-associated toxicities were radiation dermatitis and breast fibrosis. One patient reported grade 3 breast and chest wall pain post-treatment, though this was unrelated to radiotherapy (i.e. gastro-intestinal surgery for volvulus). Consequently, no radiotherapy-associated grade ≥ 3 was observed.

### Patient experience

The total treatment duration was acceptable in 87 % (65/75), and 99 % (73/74) of the patients would prefer the same treatment type again according to the in-house questionnaire (Supplementary materials, Appendix G).

## Discussion

This study demonstrates the clinical implementation of oART in postoperative partial breast, whole breast, post-mastectomy, and axillary irradiation in right- and left-sided breast cancer patients. To our knowledge, this is the first study that has evaluated oART in all these targets. We are aware of the unequal distribution of left, right and local and locoregional treatments in our dataset and have therefore discussed them separately as much as possible. On-couch median times (range) ranged from 14.5 (10.1–29.2) minutes for the WB R to 25.8 (22.6–36.8) minutes for the WB + LN I-II L. All TP_A_ met the D98% ≥ 95 % coverage criteria for the target PTV, toxicity was mainly grade 0–1, patient satisfaction was high, and skin marks were reduced to 0–1.

Building on our first experience in 20 right-sided WBI patients, we expanded this cohort to 44 and observed similar median (range) durations of 14.5 (10.1–29.2) minutes vs. previously reported 13.8 (10.7–24.6) minutes.[6] Left-sided WBI resulted in longer median treatment times due to vDIBH (20.0 (range: 10.4–32.8) minutes). Furthermore, locoregional indications took longer than local indications due to the complexity of axillary lymph node contouring, and an increased number of necessary contour adaptations, especially with Ethos 2.0. This could be explained by a greater influence of LN position to arm setup, combined with a suboptimal contour propagation based on deformable image registration (DIR), confirmed by a previous retrospective study.[17] Despite the complexity, all indications had shorter treatment times compared to previously reported PBI durations of 34.4 minutes (median) and 31.3 minutes (mean).[9,10] In the first study, an average contouring time of 9.7 minutes was reported.[9] In the second study, VMAT was used in 50 % of the patients which significantly prolonged plan computation and therefore could be a reason for the long treatment time. However, we exclusively used VMAT for PBI treatment and still achieved notably lower median (range) on-couch times of 18.9 (14.2–27.0) minutes and 20.5 (15.5–26.5) minutes for right- and left-sided, respectively. And with the exception of WBI R + LN I-IV, our median contouring times were considerably lower, which may be explained because of the omission of influencer structures.

Based on studies on intrafraction motion, we assumed that longer contouring time would increase patient displacement.[18,19] However, couch shift data based on CBCT1-CBCT2 registration, with a mean vector of 0.27 cm showed a negligible correlation between time and patient displacement. Therefore, it does support the necessity of the couch shift based on CBCT2 regardless of adaptation speed.

Consistent with prior oART studies in breast and prostate cancer, TP_A_ showed significantly improved CTV and PTV coverage (D98% ≥ 95 %) compared to TP_S_.[6,9,10,20] However, since patients were positioned without skin marks, this could not be compared to our IGRT workflow. CTV underdosage in TP_S_ could result from setup differences rather than plan quality. Consequently, it was not possible to evaluate the clinical impact based on how often an offline replanning would have been necessary without oART. Nonetheless, three causes of target underdosing in TP_S_ compared to TP_A_ were identified: differences in body contour at beam entry near the shoulder, breast contour/volume variations, and online adapted CTV expansions exceeding three slices. The first two can also occur during IGRT workflows.

A decrease in breast CTV volume was observed during the first fraction compared to the pCT, caused by a reduced postoperative seroma in the tumor bed. An increase in breast CTV volume in following fractions can be attributed predominantly to radiation-induced edema.[[21], [22], [23]] These volume changes were both observed in fractions with and without manual adaptations, indicating the contour propagation was able to deform to volume changes generally well and that manual adaptations were mainly needed for contour finetuning.

The observed toxicity was mild and consistent with other breast irradiation studies.[7,[24], [25], [26], [27], [28]] Two of our first locoregional (axillary LN I-IV) patients, however, experienced radiation dermatitis on their back, which was traced to a suboptimal beam configuration. After adjusting the fifth beam orientation from posterior-anterior to anterior-posterior, no further skin reactions occurred. In addition, after the 5th locoregional patient, we introduced a 10-field static IMRT-technique for locoregional indications to further reduce the high dose outside the PTV. In general, patients were satisfied with the treatment and would prefer the same treatment again, although most patients were not able to compare this by lack of previous radiation experience.

This study contained several limitations including dataset heterogeneity by introducing workflow developments such as expanding to our satellite location, removing the influencer structure, and transitioning to RTT-only. Also, direct comparison of oART and IGRT workflows both in terms of dosimetry and total treatment time was not performed because of incomplete and inaccurate time registrations on patients entering and exiting the room. We therefore reported the on-couch treatment times. Additionally, the number of patients treated with axillary irradiation was limited also preventing the implementation of an RTT-only workflow for these patients.

Future research will focus on implementing the RTT-only workflow in all RT targets in breast cancer patients, and identifying breast cancer patients most likely to benefit from oART. Particularly those at risk for offline replanning or setup challenges leading to extra CBCTs or suboptimal dose coverage in standard IGRT workflow. Based on adaptation frequency obtained in this study, we predict that oART, when improved target propagation is achieved, will be most beneficial in locoregional indications. In addition, investigating the clinical dosimetric relevance of the performed adaptations and evaluating real-time intrafraction motion using surface tracking could be valuable. This may help to determine whether time can be saved by less contour adaptations, or the OARs can be spared better by reducing the PTV-margins.

## Conclusion

Challenges in accurate target positioning in breast cancer radiotherapy can be addressed by online adaptive radiotherapy (oART). The implementation of oART was safe for partial breast, whole breast, and axillary lymph nodes levels in postoperative breast cancer radiotherapy. Median on-couch time was acceptable for patients and an RTT-only workflow was successfully implemented. Future work will focus on the reduction of manual contour adaptations in order to further reduce treatment times, and to optimize patient selection for oART.

## Disclosure

This research was funded by Varian Medical Systems, Inc. (Varian). Wilko F.A.R. Verbakel has received honoraria/travel expenses from Varian that are not related to the current work. Wilko F.A.R. Verbakel has been employed by both Varian and the Amsterdam UMC since May 2023.

## Declaration of Competing Interest

The authors declare that they have no known competing financial interests or personal relationships that could have appeared to influence the work reported in this paper.
