# Supplementary material for: Cone beam computed tomography-guided online adaptive radiation therapy: Clinical implementation in breast and axillary target volumes
Source: Clin Transl Radiat Oncol. 2025 Nov 27;56:101086. doi: 10.1016/j.ctro.2025.101086 (PMC12704200; doi:10.1016/j.ctro.2025.101086)
Supplement: Supplementary Data 1 [file mmc1.docx]

**Supplementary materials**

**Appendix A**

Amount of CBCTs needed for correct patient positioning

In-house data on the number of CBCTs needed to correctly position a breast cancer patient. This data was gathered over a 6-month period ranging from November 2022 until April 2023.

Table A.1-a CBCT use for local and locoregional patients in number of patients

|  | **Total in number of patients** | **>1 CBCT** | **>1 CBCT [%]** |
| --- | --- | --- | --- |
| Local | 42 | 19 | 45% |
| Locoregional | 26 | 21 | 81% |
| Total | 68 | 40 | 59% |

Table A.1-b CBCT use for local and locoregional patients in number of fractions

|  | **Total in number of fractions** | **>1 CBCT** | **>1 CBCT [%]** |
| --- | --- | --- | --- |
| Local | 380 | 66 | 17% |
| Locoregional | 405 | 79 | 20% |
| Total | 785 | 145 | 18% |

Table A.1: Overview of CBCT use for local and locoregional patients a) presented in number of patients. If ticked >1 CBCT, this means in one or more fractions of the patient’s treatment, two or more CBCTs were needed to correctly position the patient. b) Overview presented in number of fractions. Local patients were: WB ((a) n=36, (b) n=260) and WB + boost ((a) n=6, (b) n=120). Locoregional patients were: WB + LN lvl I-II ((a) n=5, (b) n=75), WB + LN lvl I-IV ((a) n=12, (b) n=180), chest wall + LN lvl I-IV ((a) n=4, (b) n=60), WB + LN lvl I-IV + boost ((a) n=3, (b) n=60), chest wall + LN lvl I-IV + parasternal ((a) n=1, (b) n=15), and WB + LN lvl I-IV + LN IMN ((a)n=1, (b) n=15)
Abbreviations: CBCT = Cone Beam Computed Tomography, WB = Whole breast, LN = Lymph nodes, IMN = internal mammary nodes.

**Appendix B**

Beam setup local and locoregional indications

The beam setup for local and locoregional (first 5 patients) indications were optimized per patient by an in-house developed script and linked to Eclipse (Varian, a Siemens Healthineers Company). The concept is as follows:

- A medio-lateral (ML) beam is positioned between 30° and 60° (Field 1 in Figure B.1).
- A latero-medial (LM) beam is positioned opposite between 213° and 243° (Field 3 in Figure B.1).
- A cost function is utilized to determine the specific angles of the ML beam and LM beam that minimizes the OAR dose while ensuring adequate PTV coverage, aiming for PTV D98% ≥ 95%.
- Contoured OARs were contralateral breast + 3 mm (to accommodate for breathing), ipsilateral lung, heart and liver.
- The position of the isocenter is chosen such that the isocenter to skin distance is the same for the opposing beams and the distance to the lung contour is approximately 7 mm.
- Two additional beams are added 15° from the ML and LM (Field 2 and Field 4 in Figure B.1).
- For a 5-beam setup, a fifth beam is added at 190° and at least a 15° distance from the fourth field. This could lead to a 181° angle at most.

The final beam configuration is exported from Eclipse to the Ethos treatment planning system, where dose optimization is carried out based on the template.


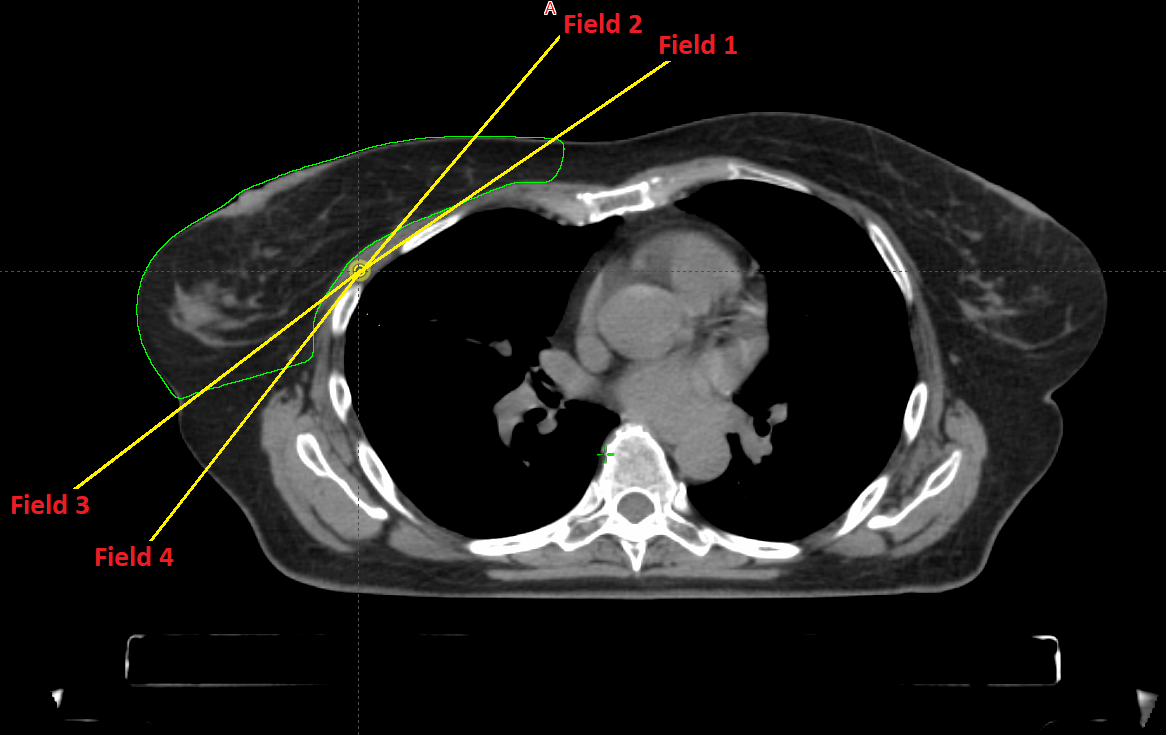


Figure B.1: 4-beam setup for right-sided local indication.

For left-sided indications the beam angles are rotated 90°. The ML beam is angled at 300-330°, the LM beam at 123-153° and the 5th beam at 170°.

Updated 10-beam setup for locoregional indications

The beam setup is scripted and linked to Eclipse (Varian, a Siemens Healthineers Company). The setup is as follows:

Right-sided WBI/PMRT+axillary LN I-IV

| Beam # | Fields | Collimator |
| --- | --- | --- |
| 1 | 30-60**°** (ML script)* | 350 |
| 2 | Beam 1 -10**°** | 355 |
| 3 | Beam 2 -10**°** | 358 |
| 4 | Beam 3 -10**°** | 7 |
| 5 | 5° ** | 358 |
| 6 | 213-243**°** (LM script)* | 10 |
| 7 | Beam 6 -10**°** | 5 |
| 8 | Beam 6 +10**°** | 3 |
| 9 | Beam 8 +10**°** | 10 |
| 10 | Beam 9 +10**°** | 8 |

*Beam positioning as described above in ‘beam setup local and locoregional indications’.
** In case beam 4 is within 5**°** of beam 5, beam 4 is either omitted or is equal to: beam 7 -10**°.**

Left-sided WBI/PMRT+axillary LN I-IV

| Beam # | Fields | Collimator |
| --- | --- | --- |
| 1 | 300-330° (ML script)* | 10 |
| 2 | Beam 1 +10**°** | 5 |
| 3 | Beam 2 +10**°** | 2 |
| 4 | Beam 3 +10**°** | 353 |
| 5 | 355° ** | 2 |
| 6 | 123-153° (LM script)* | 350 |
| 7 | Beam 6 +10**°** | 355 |
| 8 | Beam 6 -10**°** | 357 |
| 9 | Beam 8 -10**°** | 350 |
| 10 | Beam 9 -10**°** | 352 |

*Beam positioning as described above in ‘beam setup local and locoregional indications’.
** In case beam 4 is within 5**°** of beam 5, beam 4 is either omitted or is equal to: beam 7 +10**°.**


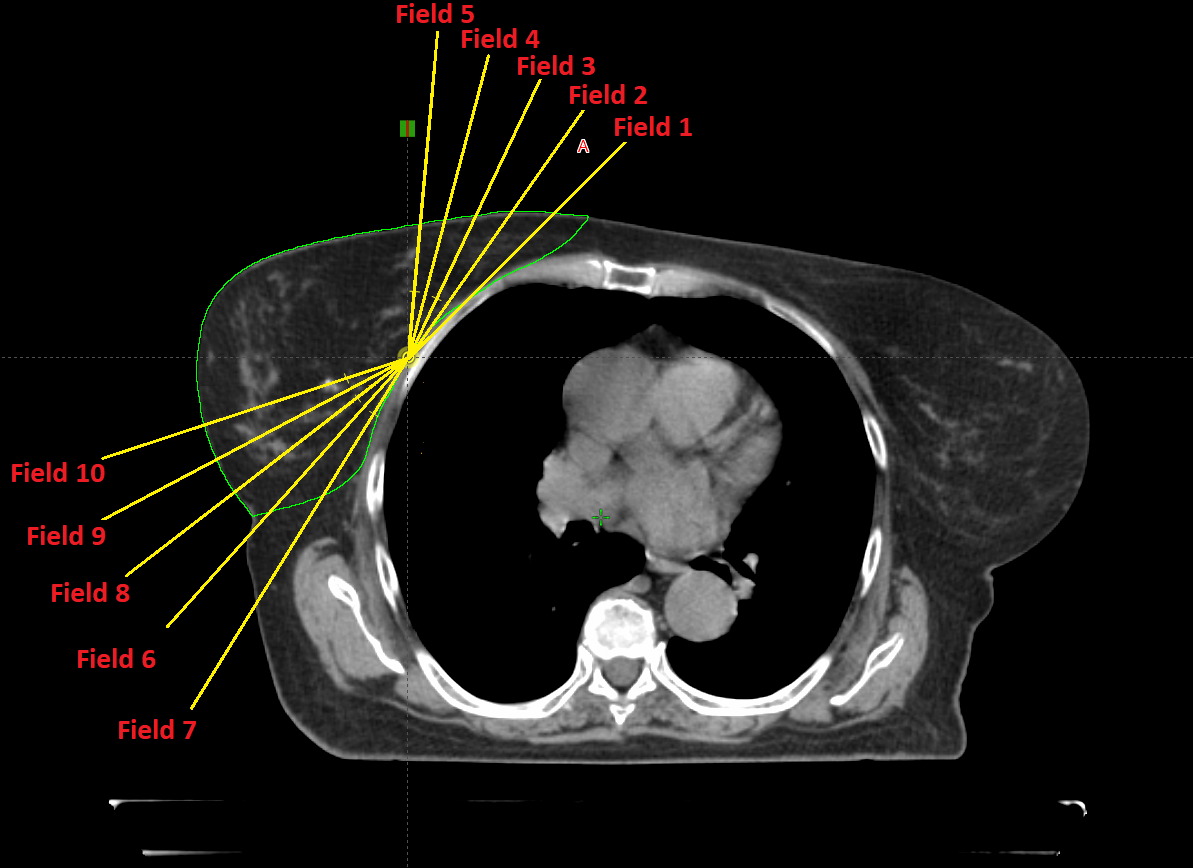


Figure B.2: 10-field setup for right-sided locoregional indication.

Mini-bolus

Ethos does not allow for a skin-flash technique. However, in order to force the optimizer to open up the MLCs a little further and create a margin to account for breathing, a mini-bolus structure is added to the body contour during treatment planning. The structure is delineated by an in-house developed script. It is drawn in the axial slice containing the isocenter by contouring a 3 voxel-wide rectangular structure perpendicular to the gantry angle of the first beam. (Figure B.3-a) The length is determined by the distance between the isocenter and the beam source, namely 1/16^th^ of this distance inwards and 1/8^th^ of this distance outwards from the isocenter. This rectangular contour is then copied to all slices from 4 slices below the target until 4 slices above the target. (Figure B.3-b) Hereafter, it is cropped such that it protrudes just 55 mm outside the body and 5 mm inside. (Figure B.4-a, B.4-b) No density is assigned. Finally, the structure is embedded in the body contour. This protruding contour creates a 3 mm margin in ventrolateral direction.


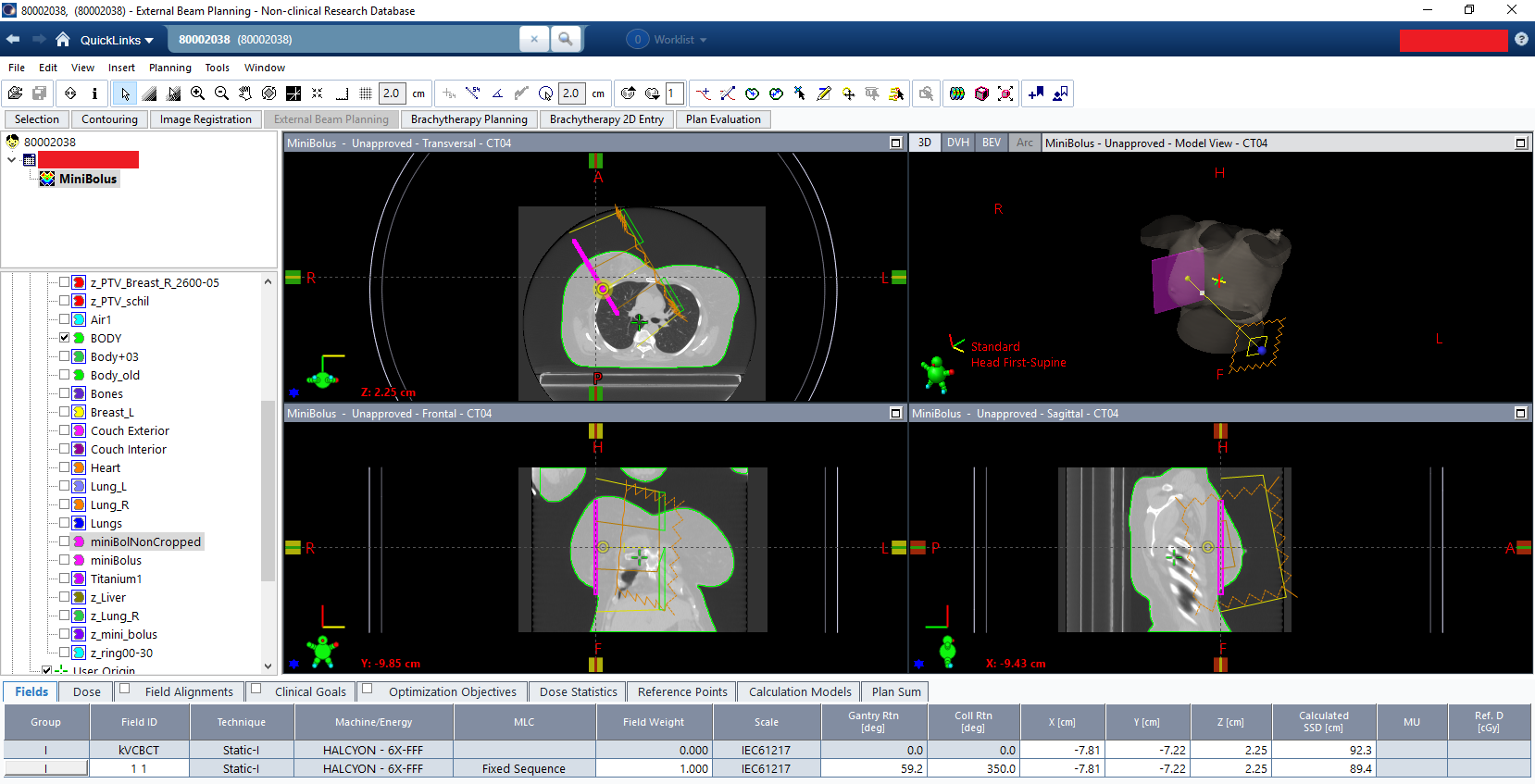

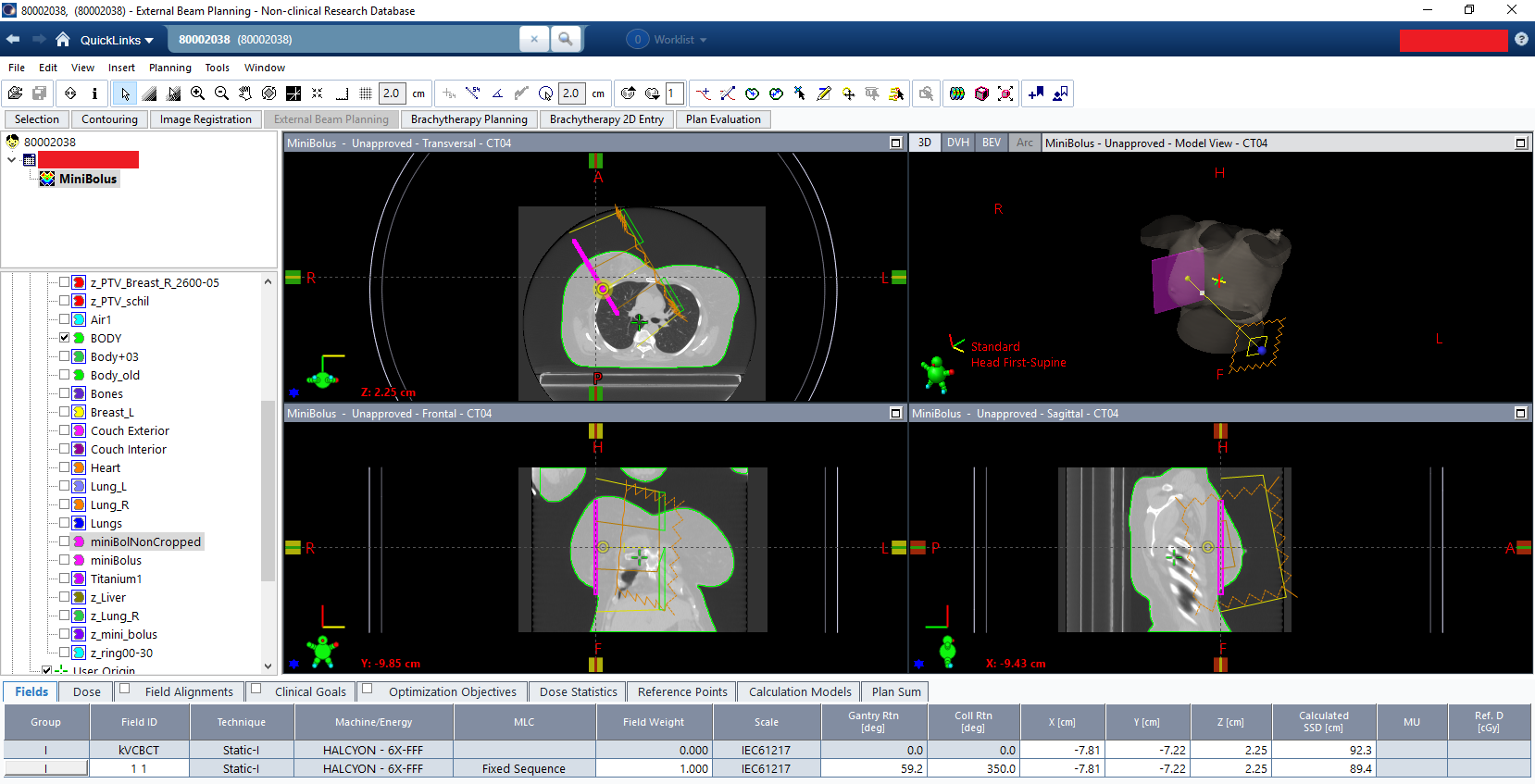


**a**

**b**.

Figure B.3: Axial view (B.3-a) of the uncropped mini-bolus arising from the isocenter and into the direction perpendicular to the gantry angle of the first beam. B.3-b shows a 3D view of the mini-bolus copied to all slices from 4 slices below the target until 4 slices above the target.


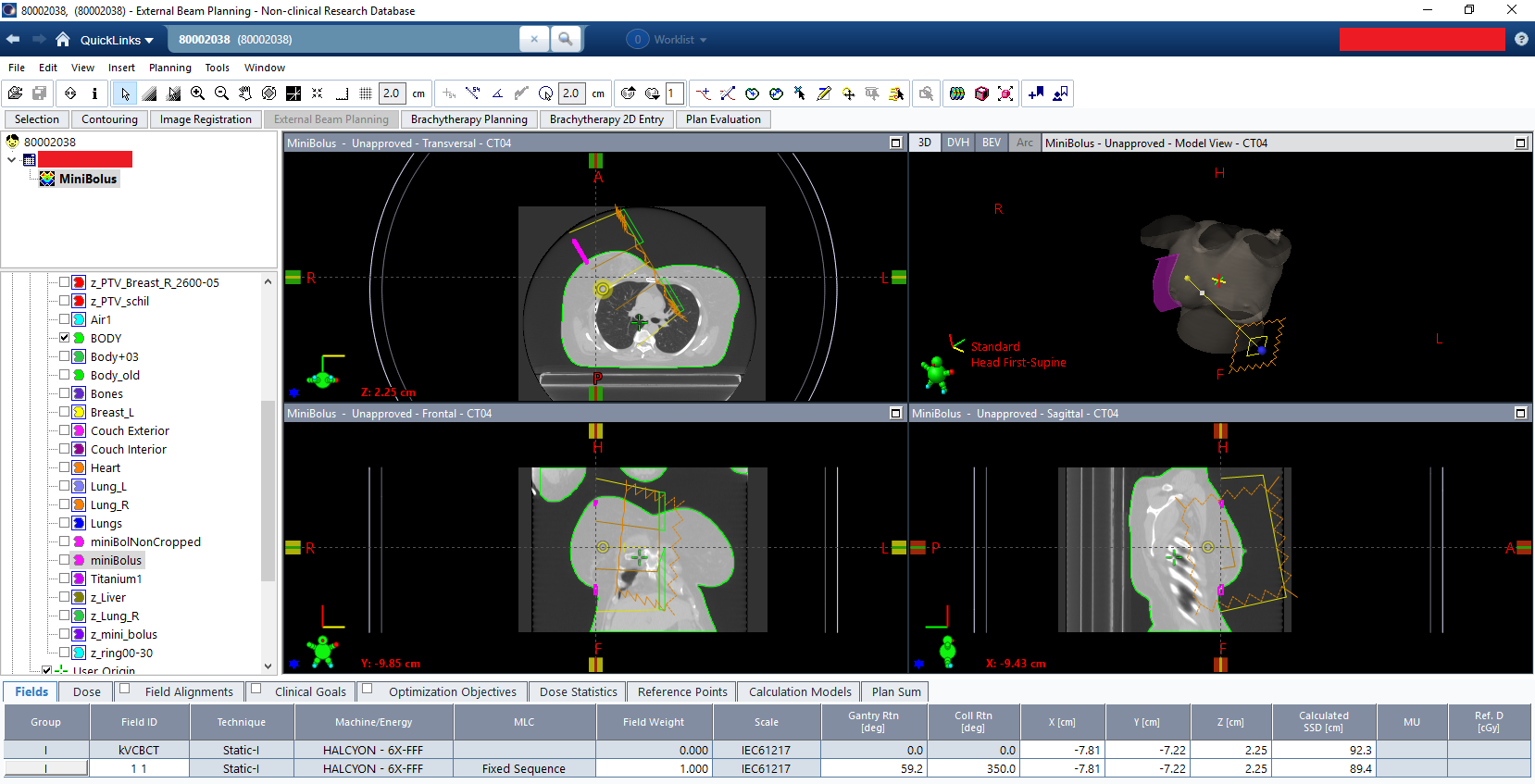

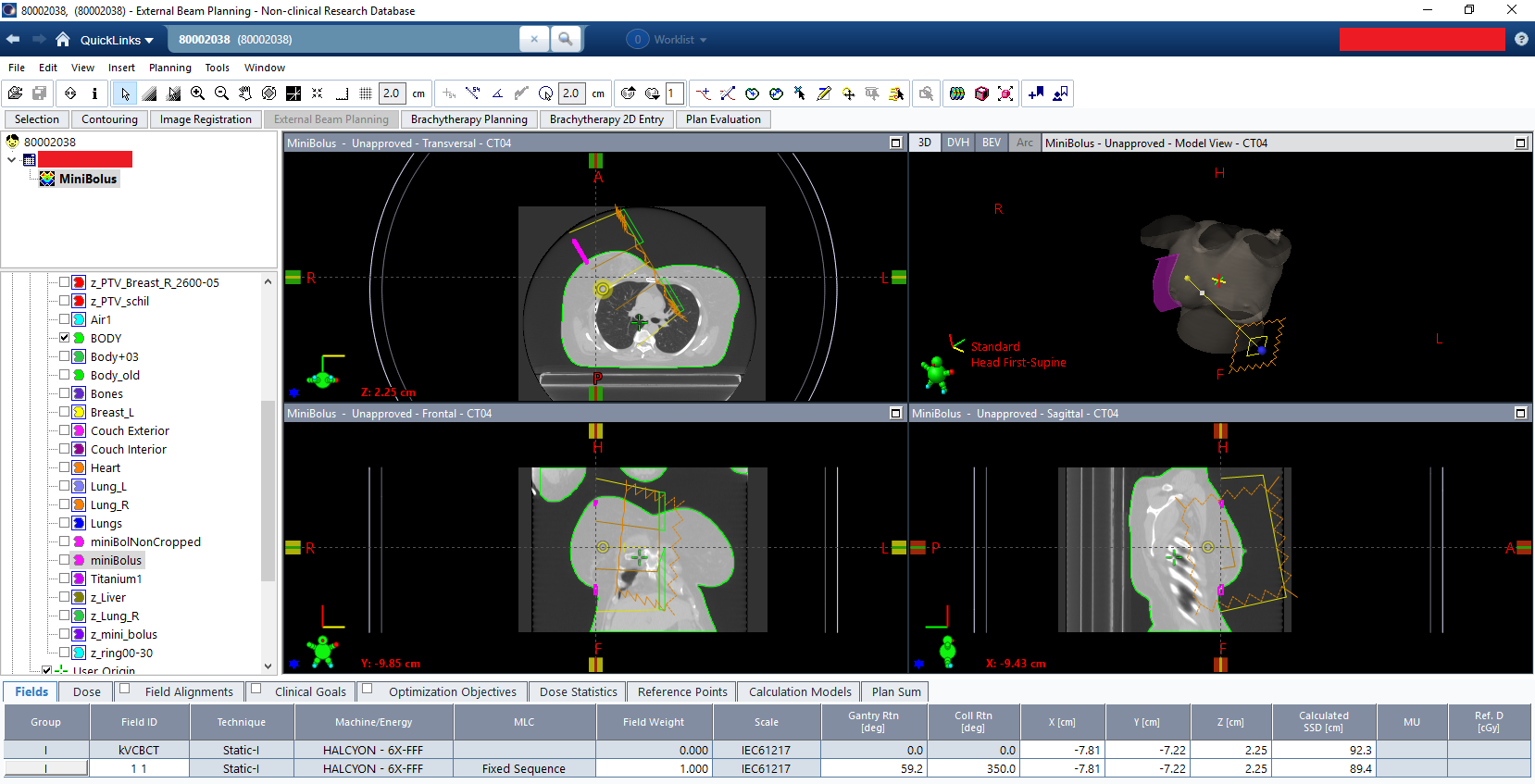


**a**

**b**.

Figure B.4: Axial view (B.4-a) and 3D view (B.4-b) of the cropped mini-bolus such that it protrudes 55 mm outside the body and 5mm inside.

Beam-setup for PBI indications

The planning technique utilized for partial breast irradiation (PBI) is VMAT. The treatment setup comprises two arcs, with a minimum arc angle of 180°. Although the arc angle may be increased to enhance modulation capabilities, care must be taken to minimize the volume of lung tissue exposed to irradiation. The two arcs are configured to rotate in opposing directions.

Virtual bolus

Normally, during plan optimization, a virtual bolus is used (for peripherally located target volumes), which is removed during final dose calculation. In Ethos, the removal during final dose calculation is not possible. It stays on during plan calculation, while in reality there is no bolus on the patient. This could lead to inaccurate dosimetry however, after testing, the following method provided clinically suitable plans with acceptable/minor differences during the evaluation of clinical goals. In this method, a virtual bolus was added during planning in Ethos by expanding the PTV and body with 5 mm and subsequently overriding this with a water density.

Template

| **Structure** | **WB R + L** | **WB + LN I-II R + L** | **WB + LN I-IV R + L** |
| --- | --- | --- | --- |
| **PTV_Breast-05** | D98% ≥ 95% | “ ” | “ ” |
|  | D2% ≤ 107% | “ ” | “ ” |
|  | D0.1cm^3^ ≤ 107% | “ ” | “ ” |
|  | V105% ≤ 4% | “ ” | “ ” |
| **PTV_LN_Ax-05** | X | D98% ≥ 95% | “ ” |
|  | X | D2% ≤ 107% | “ ” |
|  | X | D0.1cm^3^ ≤ 107% | “ ” |
|  | X | V105% ≤ 4% | “ ” |
| **Contralateral breast** | Dmean ≤ 300 cGy | “ ” | “ ” |
|  |  | D0.1% ≤ 1950 cGy | D0.1% ≤ 3000 cGy |
| **Heart** | Dmean ≤ 80 cGy | Dmean ≤ 100 cGy (200 cGy*) | “ ” |
| **Ipsilateral Lung** | V5Gy ≤ 20% | V5Gy ≤ 50% | “ ” |
|  | V20Gy ≤ 10% | V20Gy ≤ 25% | “ ” |
| **Lungs** | Dmean ≤ 300 cGy | Dmean ≤ 500 cGy | “ ” |
| **Ipsilateral BrachialPlex** | X | D0.1cm^3^ ≤ 4005 cGy | “ ” |
| **Thyroid Gland** | X | V3000 cGy ≤ 50% | “ ” |
| **Z_ring-30** | D1cm^3^ ≤ 2782 cGy | X | X |
|  | D0.03 cm^3^ ≤ 2860 cGy | X | X |
| **Z_ring-50** | X | D1cm^3^ ≤ 2730 cGy | D1cm^3^ ≤ 4205 cGy |
|  | X | D0.1 cm^3^ ≤ 2860 cGy | D0.1 cm^3^ ≤ 4406 cGy |
| **Z_ring_sternum** | X | X | D1cm^3^ ≤ 3604 cGy |
|  | X | X | D0.1cm^3^ ≤ 3604 cGy |

*Left-sided

| **Structure** | **PBI R + L** |
| --- | --- |
| **PTV_2600** | D2% ≤ 107% |
| **PTV_2600-05** | D98% ≥ 95% |
|  | Dmean ≥ 98% |
|  | Dmean ≤ 102% |
|  | D0.1cm^3^ ≤ 107% |
| **CTV** | V100% ≥ 60% |
| **Contralateral breast** | Dmean ≤ 100 cGy |
| **Heart** | Dmean ≤ 50 cGy |
| **Ipsilateral lung** | V5Gy ≤ 10%* |
| **Lungs** | Dmean ≤ 300 cGy |
| **Mediastinum** | Dmean ≤ 50 cGy |
| **Liver/bowel** | Dmean ≤ 50 cGy |

*Is optimized per patient to reach the lowest possible lung dose, without losing plan quality

**Appendix C**

Flowchart representing the developments for all indications and targets at both locations at the Amsterdam UMC.

**
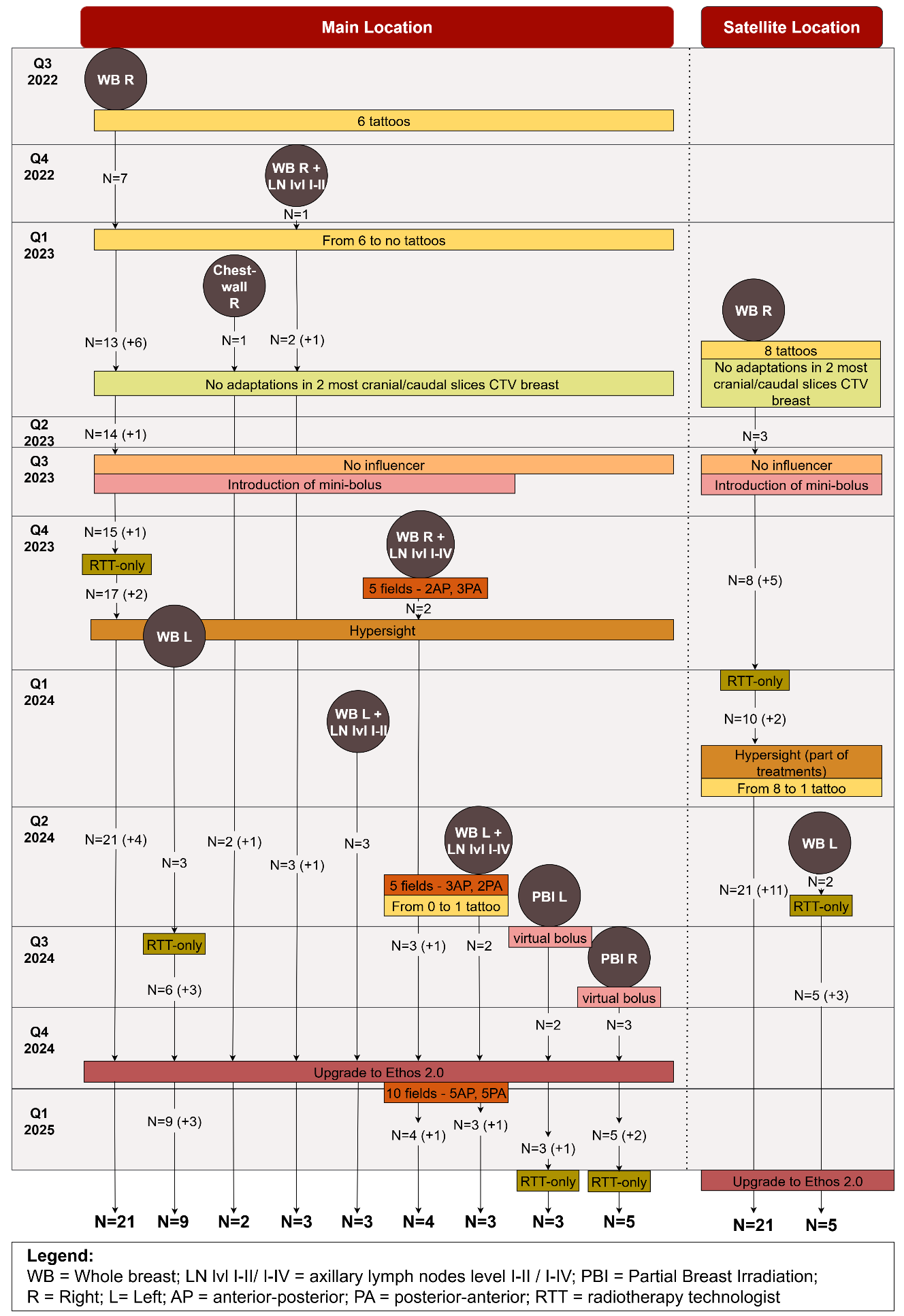
**

**Appendix D**

Total median (range) treatment times in minutes for each indication. Entering and exiting were registered manually (column ‘Enter + CBCT1’ and ‘Enter-Exit’) and therefore could be inaccurate. In addition, there is data missing in 48/425 fractions. All other times were extracted from DICOM data (N=425) and are complete, except for the column ‘treatment + CBCT3’ which has data missing in 14/425 fractions.

|  | Enter + CBCT1 | End CBCT1 + sCT gen | Contour-ing | Plan Calc | Plan approve + CBCT 2 | Treatment + CBCT 3 | Total CBCT1-CBCT3 | Enter-Exit |
| --- | --- | --- | --- | --- | --- | --- | --- | --- |
| WB R | 4.0 (0.2-32.1) | 1.2 (0.9-1.7) | 2.7 (0.4-16.6) | 1.1 (0.8-1.4) | 3.8 (2.1-8.3) | 5.3 (4.0-17.5) | 14.5 (10.1-29.2) | 20 (10-48) |
| WB L | 5.2 (0.5-62.7) | 1.2 (0.9-1.5) | 3.4 (0.6-10.1) | 1.3 (1.0-2.1) | 4.1 (2.5-9.9) | 10.3 (3.8-17.4) | 20.0 (10.4-32.8) | 28 (13-40) |
| WB + LN I-II R | 3 (1.6-11.1) | 1.1 (0.9-1.7) | 4.4 (0.8-10.2) | 1.3 (1.3-1.5) | 4.2 (3.1-6.9) | 6.1 (5.1-7.0) | 17.5 (14.5-24.1) | 22.5 (18-39) |
| WB + LN I-II L | 6.8 (3.4-10.6) | 1.4 (1.3-1.6) | 5.9 (4.5-16.3) | 1.5 (1.4-1.7) | 3.7 (3.2-4.6) | 13.0 (9.6-16.5) | 25.8 (22.6-36.8) | 33.5 (30-42) |
| WB + LN I-IV R | 4.5 (0.2-11) | 1.4 (1.0-1.7) | 10.7 (1.1-25.8) | 1.7 (0.0-3.5) | 4.4 (3.4-8.1) | 6.0 (4.4-9.5) | 25.2 (14.1-42.4) | 31 (16-50) |
| WB + LN I-IV L | 5.5 (3-13.6) | 1.4 (1.0-1.6) | 6.4 (0.8-14.3) | 1.8 (1.6-3.3) | 4.4 (3.3-5.6) | 10.4 (8.2-14.9) | 25.1 (16.7-34.3) | 32 (21-44) |
| PBI R | 3.8 (0.7 – 8.9) | 1.2 (0.8-1.4) | 2.8 (0.1-8.0) | 7.3 (6.2-10.0) | 2.8 (2.2-3.9) | 4.1 (3.3-8.2) | 18.9 (14.2-27.0) | 24 (19 – 30) |
| PBI L | 5.6 (2.8-8.6) | 1.0 (0.8-1.4) | 0.4 (0.1-7.7) | 10.2 (7.4-11.5) | 2.8 (2.0-3.3) | 4.5 (3.5-7.2) | 20.5 (15.5-26.5) | 27 (21-33) |

**Appendix E**

Figure E.1: The time between CBCT1 and CBCT2 and the length vector of the couch shift performed after CBCT2. A very weak significant positive correlation was found between time and the length vector of the performed couch shifts (ρ(465)=0.196, *P*<.001).

**Appendix F**

Extended dosimetric results of target goals and OAR constraints.

**WB R**

|  | **CTV_Breast-05** | **PTV_Breast-05** | **PTV_Breast-05** | **PTV_Breast-05** | **PTV_Breast-05** |
| --- | --- | --- | --- | --- | --- |
|  | D98% ≥ 95% | D98% ≥ 95% | D2% ≤ 107% | D0.1cm^3^ ≤107% | Dmean % |
| **TP_R_** | 96.8 (94.8-97.9) | 95.7 (95.0-97.1) | 103.7 (102.9-104.3) | 107.5 (104.8-112.9) | 100.1 (99.1-101.0) |
| **TP_S_** | 96.2 (89.6-98.1) | 94.3 (67.5-96.6) | 104.0 (102.4-107.5) | 108.0 (105.2-114.6) | 99.8 (97.7-101.7) |
| **TP_A_** | 96.7 (94.6-98.8) | 95.7 (94.9-97.3) | 103.7 (102.1-104.7) | 107.4 (105.2-115.6) | 100.1 (98.7-101.4) |

|  | **Contralateral breast** | **Heart** | **Contralateral Lung** | **Contralateral Lung** | **Lungs** |
| --- | --- | --- | --- | --- | --- |
|  | Dmean ≤ 300 cGy | Dmean ≤ 80 cGy | V5Gy ≤ 20% | V20Gy ≤ 10% | Dmean ≤ 300 cGy |
| **TP_R_** | 3.3 (1.8-21.6) | 7.0 (5.0-10.6) | 17.3 (3.5-30.4) | 5.7 (0.5-18.3) | 38.4 (22.0-71.4) |
| **TP_S_** | 3 (1-28) | 7 (4-11) | 16.8 (5.4-37.7) | 4.8 (0-17.6) | 39 (21-89) |
| **TP_A_** | 3 (2-21) | 7 (4-23.7) | 17.2 (6.4-35.9) | 5 (0.3-15.9) | 38 (22-85) |

**WB L**

|  | **CTV_Breast-05** | **PTV_Breast-05** | **PTV_Breast-05** | **PTV_Breast-05** | **PTV_Breast-05** |
| --- | --- | --- | --- | --- | --- |
|  | D98% ≥ 95% | D98% ≥ 95% | D2% ≤ 107% | D0.1cm^3^ ≤107% | Dmean % |
| **TP_R_** | 96.9 (96-98.1) | 95.7 (95.2-97.5) | 104 (102.8-104.7) | 109 (107.5-110.7) | 100.6 (99-101.6) |
| **TP_S_** | 96.3 (93.7-97.3) | 94.4 (75.5-96.6) | 104.2 (103-105.8) | 109.1 (106.7-113.3) | 100 (98.4-101.1) |
| **TP_A_** | 96.9 (95.8-98.1) | 95.8 (95.1-97.6) | 104 (102.7-105.2) | 108.7 (106.6-116.4) | 100.6 (98.9-101.8) |

|  | **Contralateral breast** | **Heart** | **Contralateral Lung** | **Contralateral Lung** | **Lungs** |
| --- | --- | --- | --- | --- | --- |
|  | Dmean ≤ 300 cGy | Dmean ≤ 80 cGy | V5Gy ≤ 20% | V20Gy ≤ 10% | Dmean ≤ 300 cGy |
| **TP_R_** | 3 (2-7) | 11.5 (9-20) | 16.1 (6.3-23.7) | 5.4 (2-18.8) | 33.5 (22-46.8) |
| **TP_S_** | 3 (2-8) | 11.5 (7-22) | 15.6 (7.3-23.8) | 4.7 (0.8-7.7) | 31 (18-47) |
| **TP_A_** | 3 (2-8) | 11 (8-21) | 16.4 (10.0-23.1) | 4.9 (2.1-7.8) | 32.5 (22-47) |

**WB + LN I-II R**

|  | **CTV_Breast-05** | **PTV_Breast-05** | **PTV_Breast-05** | **PTV_Breast-05** | **PTV_Breast-05** |
| --- | --- | --- | --- | --- | --- |
|  | D98% ≥ 95% | D98% ≥ 95% | D2% ≤ 107% | D0.1cm^3^ ≤107% | Dmean % |
| **TP_R_** | 97.5 (96.6-97.9) | 97.1 (95.9-97.2) | 103.9 (103.9-104.2) | 107.2 (106.8-107.5) | 101 (100-101.5) |
| **TP_S_** | 97.3 (93.1-98.3) | 96.4 (92.8-97.6) | 104.6 (103.8-106.1) | 108.1 (106.9-112.4) | 100.9 (99.7-101.8) |
| **TP_A_** | 97.9 (96.3-98.3) | 97.1 (95.6-97.5) | 104.2 (103.9-105) | 107.4 (106-109) | 101.1 (99.9-101.5) |

|  | **CTV_LN_Ax-05** | **PTV_LN_Ax-05** | **PTV_LN_Ax-05** | **PTV_LN_Ax-05** | **PTV_LN_Ax-05** |
| --- | --- | --- | --- | --- | --- |
|  | D98% ≥ 95% | D98% ≥ 95% | D2% ≤ 107% | D0.1cm^3^ ≤ 107% | Dmean % |
| **TP_R_** | 97.5 (96.9-97.5) | 96.6 (96.2-96.8) | 103.9 (102.7-104.1) | 105.8 (104.7-106.8) | 99.8 (99.2-100.2) |
| **TP_S_** | 96.5 (90.4-98.8) | 94.3 (79.5-97) | 103.7 (100.8-104.9) | 105.9 (102.8-107) | 99.4 (97.3-101) |
| **TP_A_** | 97.3 (96.9-97.7) | 96.6 (96.2-96.9) | 103.7 (102.6-104.4) | 105.8 (104.2-107.7) | 99.7 (99.1-100.2) |

|  | **Contralateral breast** | **Heart** | **Contralateral Lung** | **Contralateral Lung** | **Lungs** |
| --- | --- | --- | --- | --- | --- |
|  | Dmean ≤ 300 cGy | Dmean ≤ 100 cGy | V5Gy ≤ 50% | V20Gy ≤ 25% | Dmean ≤ 500 cGy |
| **TP_R_** | 4.8 (4.2-5.2) | 6.2 (6-12) | 26.9 (25.7-45.1) | 8.4 (6.7-15.1) | 56.4 (44.2-82.6) |
| **TP_S_** | 6 (4-8) | 8 (6-12) | 26.1 (21.5-43.6) | 7.5 (6-14.3) | 56 (48-81) |
| **TP_A_** | 5 (4-10) | 8 (6-13) | 25.7 (21.1-46.4) | 6.8 (5.9-15.6) | 56 (45-85) |

**WB + LN I-II L**

|  | **CTV_Breast-05** | **PTV_Breast-05** | **PTV_Breast-05** | **PTV_Breast-05** | **PTV_Breast-05** |
| --- | --- | --- | --- | --- | --- |
|  | D98% ≥ 95% | D98% ≥ 95% | D2% ≤ 107% | D0.1cm^3^ ≤107% | Dmean % |
| **TP_R_** | 97.7 (96.9-97.7) | 97.1 (96.6-97.5) | 104 (103.9-104.2) | 107.2 (107-107.7) | 100.9 (100.8-101.1) |
| **TP_S_** | 96.7 (96-97.7) | 95.9 (94.8-97.3) | 103.6 (103.3-105.2) | 106.6 (105.4-109.2) | 100.2 (99.6-101.5) |
| **TP_A_** | 97.7 (96.7-98.1) | 97.2 (96.4-97.7) | 104 (103.7-104.2) | 107.4 (106.5-108.5) | 100.9 (100.3-101.4) |

|  | **CTV_LN_Ax-05** | **PTV_LN_Ax-05** | **PTV_LN_Ax-05** | **PTV_LN_Ax-05** | **PTV_LN_Ax-05** |
| --- | --- | --- | --- | --- | --- |
|  | D98% ≥ 95% | D98% ≥ 95% | D2% ≤ 107% | D0.1cm^3^ ≤ 107% | Dmean |
| **TP_R_** | 97.4 (97.3-97.7) | 96.5 (96.4-96.9) | 103.7 (103.7-103.8) | 105.7 (105.5-106.4) | 100 (99.8-100.2) |
| **TP_S_** | 96.5 (92.9-98.8) | 93.6 (91.7-97.7) | 103.2 (102.1-105) | 105.9 (104.3-107.7) | 99 (97-100.8) |
| **TP_A_** | 97.5 (96.9-97.7) | 96.8 (96.2-96.9) | 103.8 (101.4-104.1) | 105.9 (103.4-107.1) | 100 (98.8-100.4) |

|  | **Contralateral breast** | **Heart** | **Contralateral Lung** | **Contralateral Lung** | **Lungs** |
| --- | --- | --- | --- | --- | --- |
|  | Dmean ≤ 300 cGy | Dmean ≤ 100 cGy | V5Gy ≤ 50% | V20Gy ≤ 25% | Dmean ≤ 500 cGy |
| **TP_R_** | 5 (4-8.8) | 15 (13-19.2) | 26.9 (26.4-31.5) | 8.9 (8.5-9.8) | 50 (40-55.2) |
| **TP_S_** | 6 (4-9) | 17 (12-25) | 26.8 (22.4-34.9) | 7.6 (6.5-11) | 45 (37-61) |
| **TP_A_** | 5 (5-7) | 17 (12-20) | 27.2 (23.8-36.3) | 8.4 (7.2-8.9) | 46 (39-59) |

**WB + LN I-IV R**

|  | **CTV_Breast-05** | **PTV_Breast-05** | **PTV_Breast-05** | **PTV_Breast-05** | **PTV_Breast-05** |
| --- | --- | --- | --- | --- | --- |
|  | D98% ≥ 95% | D98% ≥ 95% | D2% ≤ 107% | D0.1cm^3^ ≤107% | Dmean % |
| **TP_R_** | 97.2 (94.4-98.1) | 97.2 (96.4-97.4) | 104.3 (104.1-104.6) | 110.1 (107.5-111.1) | 101.0 (100.8-101.6) |
| **TP_S_** | 96.3 (91-98.1) | 95.4 (86.2-97.3) | 104.2 (103.1-105.3) | 109.2 (98-112.3) | 100.2 (98.4-101.6) |
| **TP_A_** | 96.6 (94.3-98.5) | 97.0 (95.6-97.7) | 104.0 (103.3-104.6) | 110.0 (105.9-113.2) | 100.8 (99.6-101.7) |

|  | **CTV_LN_Ax-05** | **PTV_LN_Ax-05** | **PTV_LN_Ax-05** | **PTV_LN_Ax-05** | **PTV_LN_Ax-05** |
| --- | --- | --- | --- | --- | --- |
|  | D98% ≥ 95% | D98% ≥ 95% | D2% ≤ 107% | D0.1cm^3^ ≤ 107% | Dmean % |
| **TP_R_** | 97.2 (96.6-97.4) | 96.6 (95.9-96.8) | 104.3 (104.1-104.6) | 107.5 (106.7-108.2) | 100.1 (100-100.5) |
| **TP_S_** | 95.5 (88.4-97.8) | 91.2 (61.2-96.1) | 103.9 (100.7-106.6) | 107.3 (104.5-114.2) | 98.7 (95-101.8) |
| **TP_A_** | 97.0 (95.5-97.8) | 96.5 (95.1-97.1) | 104.1 (103.7-105) | 107.0 (105.9-109.7) | 100.0 (99.7-100.5) |

|  | **Contralateral breast** | **Heart** | **Contralateral Lung** | **Contralateral Lung** | **Lungs** |
| --- | --- | --- | --- | --- | --- |
|  | Dmean ≤ 300 cGy | Dmean ≤ 100 cGy | V5Gy ≤ 50% | V20Gy ≤ 25% | Dmean ≤ 500 cGy |
| **TP_R_** | 5.1 (2.6-8.3) | 5.2 (4-7.1) | 43.1 (39.2-50.1) | 20.9 (17.2-25.4) | 35.5 (29.9-47.2) |
| **TP_S_** | 7 (2-13) | 6 (4-8) | 43.4 (37.3-49.8) | 20.2 (17.2-25.1) | 39 (33-46) |
| **TP_A_** | 7 (2-13) | 6 (4-8) | 43.2 (35.5-59.3) | 19.8 (14.8-26.5) | 38 (30-49) |

**WB + LN I-IV L**

|  | **CTV_Breast-05** | **PTV_Breast-05** | **PTV_Breast-05** | **PTV_Breast-05** | **PTV_Breast-05** |
| --- | --- | --- | --- | --- | --- |
|  | D98% ≥ 95% | D98% ≥ 95% | D2% ≤ 107% | D0.1cm^3^ ≤107% | Dmean % |
| **TP_R_** | 97 (96.6-97) | 96.3 (95.6-96.8) | 103.9 (103.9-105.2) | 107.2 (107.1-110) | 100.6 (100.5-100.8) |
| **TP_S_** | 95.5 (92.5-97) | 95.8 (89.4-96.8) | 105.1 (103.5-106.9) | 108.4 (106-111.1) | 100.3 (98-101.5) |
| **TP_A_** | 96.6 (95.9-97.4) | 96.5 (96.2-97.1) | 104.1 (103.5-105.1) | 107.4 (106.1-112.4) | 100.6 (99.8-101) |

|  | **CTV_LN_Ax-05** | **PTV_LN_Ax-05** | **PTV_LN_Ax-05** | **PTV_LN_Ax-05** | **PTV_LN_Ax-05** |
| --- | --- | --- | --- | --- | --- |
|  | D98% ≥ 95% | D98% ≥ 95% | D2% ≤ 107% | D0.1cm^3^ ≤ 107% | Dmean % |
| **TP_R_** | 97.3 (96.3-97.4) | 96.6 (95.9-96.8) | 103.8 (103.1-103.9) | 106.3 (106.1-108.2) | 100 (99.5-100.3) |
| **TP_S_** | 95.9 (80.9-98.1) | 94.4 (70-97) | 103.6 (100.1-106.4) | 106.9 (102.8-111.1) | 99.6 (93-100.8) |
| **TP_A_** | 97 (96.3-97.8) | 96.6 (95.9-97.1) | 103.5 (102.6-104) | 106.3 (104.7-108.4) | 99.9 (99.5-100.4) |

|  | **Contralateral breast** | **Heart** | **Contralateral Lung** | **Contralateral Lung** | **Lungs** |
| --- | --- | --- | --- | --- | --- |
|  | Dmean ≤ 300 cGy | Dmean ≤ 100 cGy | V5Gy ≤ 50% | V20Gy ≤ 25% | Dmean ≤ 500 cGy |
| **TP_R_** | 5 (2.8-13) | 6.4 (6-12) | 40.1 (31.6-41.4) | 16.7 (13.5-19.2) | 27.8 (24-27.8) |
| **TP_S_** | 5 (2-16) | 7 (6-16) | 38.1 (30-47.2) | 15.9 (14.3-21) | 26 (22-33) |
| **TP_A_** | 6 (2-17) | 6 (6-14) | 32.8 (30.7-45.7) | 15.8 (11.6-19.8) | 25 (22-29) |

**PBI R**

|  | **CTV-05** | **PTV-05** | **PTV-05** | **PTV-05** | **PTV-05** |
| --- | --- | --- | --- | --- | --- |
|  | D98% ≥ 95% | D98% ≥ 95% | D2% ≤ 107% | D0.1cm^3^ ≤107% | Dmean % |
| **TP_R_** | 97.5 (96.3-97.9) | 97.5 (95-97.7) | 104.2 (103.2-105.1) | 106.5 (104.6-107.9) | 101 (100-101.1) |
| **TP_S_** | 97.3 (83.8-98.8) | 95.8 (86.1-98) | 103.9 (103.1-105.8) | 106.3 (104.7-108.9) | 100.3 (99.1-101.7) |
| **TP_A_** | 97.9 (88.7-98.8) | 97.6 (95-98.1) | 104.3 (102.6-105.4) | 106.5 (103.6-108) | 101.1 (99.6-101.5) |

|  | **Contralateral breast** | **Heart** | **Contralateral Lung** | **Lungs** |
| --- | --- | --- | --- | --- |
|  | Dmean ≤ 100 cGy | Dmean ≤ 50 cGy | V5Gy ≤ 10% | Dmean ≤ 300 cGy |
| **TP_R_** | 4 (2-37) | 4 (2-15) | 4.4 (0-14.8) | 17 (7-35) |
| **TP_S_** | 4 (2-43) | 4 (1-17) | 3.8 (0-16.8) | 17 (6-38) |
| **TP_A_** | 4 (1-44) | 4 (1-15) | 2.7 (0-17.8) | 11 (5-39) |

**PBI L**

|  | **CTV-05** | **PTV-05** | **PTV-05** | **PTV-05** | **PTV-05** |
| --- | --- | --- | --- | --- | --- |
|  | D98% ≥ 95% | D98% ≥ 95% | D2% ≤ 107% | D0.1cm^3^ ≤107% | Dmean % |
| **TP_R_** | 97.9 (97.5-98.5) | 97.7 (96.6-98) | 104.8 (104.6-105) | 106.6 (106.4-106.7) | 101.6 (101.4-101.9) |
| **TP_S_** | 98.5 (96.7-99) | 97.1 (94.6-97.9) | 104.8 (104.2-105.3) | 106.3 (105.8-107.4) | 101.7 (100.2-102.5) |
| **TP_A_** | 98.1 (97.1-99.2) | 97.7 (96.3-98.5) | 104.8 (104.4-105.3) | 106.9 (106.4-107.6) | 101.6 (101-102) |

|  | **Contralateral breast** | **Heart** | **Contralateral Lung** | **Lungs** |
| --- | --- | --- | --- | --- |
|  | Dmean ≤ 100 cGy | Dmean ≤ 50 cGy | V5Gy ≤ 10% | Dmean ≤ 300 cGy |
| **TP_R_** | 13 (6-28) | 15 (10-16) | 5.8 (5.8-6.2) | 17 (14-24) |
| **TP_S_** | 14 (6-29) | 12 (9-19) | 5.2 (4-5.9) | 16 (12-22) |
| **TP_A_** | 14 (5-31) | 13 (8-17) | 5.6 (3.4-7.6) | 15 (11-25) |

**Appendix G**

Questionnaire

In-house developed questionnaire (translated from Dutch). All questions had the following answer options: 1 – not at all, 2 – a little, 3- quite, 4 – a lot. Negative outcomes or dissatisfaction was defined by scoring 1 for questions 1-3, 5-7, and 10; and by scoring 4 for questions 4, 8, and 9.

Translated questionnaire (developed in-house)

Given the intensity of treatment on this radiation device, we would like to evaluate your experience. Could you please indicate how much each of these questions applies to your experience?

Options: 1 – not at all, 2 – a little, 3- quite, 4 – a lot

1. Was the explanation of the treatment preparation and procedure understandable?
2. Did the radiation oncologist adequately inform you regarding possible side effects?
3. Was the waiting time between your first consultation with the radiation oncologist and the commencement of your treatment acceptable?
4. Did you experience anxiety during your time on the treatment table?
5. Was the amount of time spent on the treatment table acceptable?
6. Was communication during treatment understandable?
7. Did you think the radiation device's support staff was professional in their approach?
8. Did symptoms from your disease increase during the treatment?
9. Did you experience the total duration at the department as long?
10. In case you are referred for radiotherapy again, would you prefer the same treatment pathway?

Original questionnaire in Dutch (Version 2, June 16, 2022):

Vanwege de intensieve behandeling op dit bestralingsapparaat, willen wij graag evalueren hoe u dit hebt ervaren. Wilt u aangeven in welke mate onderstaande vragen op u van toepassing zijn?

Options: 1 – helemaal niet, 2- een beetje, 3 – nogal, 4 - heel erg

1. Was de uitleg over de procedure van de voorbereiding en de behandeling begrijpelijk?
2. Was u door de bestralingsarts goed voorbereid op de te verwachten bijwerkingen?
3. Was de wachttijd tussen het eerste consult bij de bestralingsarts en start van de behandeling acceptabel?
4. Voelde u zich angstig tijdens het liggen in het bestralingstoestel?
5. Was de tijd dat u op het bestralingstoestel lag acceptabel?
6. Was de communicatie tijdens de behandeling begrijpelijk?
7. Heeft u de begeleiding van het personeel op het bestralingstoestel als professioneel ervaren?
8. Zijn de klachten die u als gevolg van uw ziekte heeft, toegenomen tijdens de behandeling?
9. Heeft u de totale duur dat u op de afdeling aanwezig was als lang ervaren?
10. Indien u opnieuw verwezen wordt voor een bestraling, vind u het dan prettig om op dezelfde manier behandeld te worden?

Figure G.1: Patient experience according to the results of the in-house developed questionnaire filled in by 75/79 patients. Q8 and Q10 have 74 entries.
